# vACcine COnfidence amongst those living with alleRgy during the COVID pandemic (ACCORD): a scoping review protocol

**DOI:** 10.1186/s13223-022-00723-w

**Published:** 2022-09-18

**Authors:** Michael A. Golding, Nicole Askin, Ayel Luis R. Batac, Kaitlyn A. Merrill, Elissa M. Abrams, Philippe Bégin, Moshe Ben-Shoshan, Erika Ladouceur, Leslie E. Roos, Vladan Protudjer, Jennifer L. P. Protudjer

**Affiliations:** 1 Department of Pediatrics and Child Health, Rady Faculty of Health Sciences, Max Rady College of Medicine, University of Manitoba, Winnipeg, MB Canada; 2 Children’s Hospital Research Institute of Manitoba, 501G-715 McDermot Ave, Winnipeg, MB R3E 3P4 Canada; 3 WRHA Virtual Library, University of Manitoba, Winnipeg, MB Canada; 4 Division of Allergy and Immunology, Department of Pediatrics, Faculty of Medicine, University of British Columbia, Vancouver, BC Canada; 5 Division of Allergy, Department of Pediatrics, Centre Hospitalier Universitaire Sainte-Justine, Montreal, QC Canada; 6 Division of Allergy, Department of Medicine, Centre Hospitalier de L’Université de Montréal, Montreal, QC Canada; 7 Division of Pediatric Allergy, Clinical Immunology and Dermatology, Montreal Children’s Hospital, Montreal, QC Canada; 8 Centre for Outcomes Research and Evaluation, Research Institute of the McGill University Health Centre, Montreal, QC Canada; 9 Department of Epidemiology, Biostatistics and Occupational Health, School of Population and Global Health, Faculty of Medicine and Health Sciences, McGill University, Montreal, QC Canada; 10 Division of Experimental Medicine, Department of Medicine, School of Medicine, Faculty of Medicine and Health Sciences, McGill University, Montreal, QC Canada; 11 Science for All Audiences, Toronto, ON Canada; 12 Department of Psychology, Faculty of Arts, University of Manitoba, Winnipeg, MB Canada; 13 College of Nursing, Rady Faculty of Health Sciences, University of Manitoba, Winnipeg, MB Canada; 14 Department of Food and Human Nutritional Sciences, Faculty of Agricultural and Food Sciences, University of Manitoba, Winnipeg, MB Canada; 15 Fay Yee Centre for Healthcare Innovation, Winnipeg, MB Canada; 16 Centre for Allergy Research, Karolinska Institutet, Stockholm, Sweden

## Abstract

**Background:**

Reports of allergic reactions to the COVID-19 vaccines have been documented, which may also contribute to hesitancy. Despite the low likelihood that the COVID-19 vaccine will trigger an allergic reaction, we and others have reported that families with allergy remain vaccine hesitant due to concerns of COVID-19-vaccine-triggered anaphylaxis.

**Objective:**

To present our scoping review protocol, that will inform a forthcoming living scoping review in which we will investigate the peer-reviewed and grey literature on COVID-19 vaccine hesitancy and allergic disease and/or allergic reactions following a COVID-19 vaccine.

**Methods:**

Informed by Arksey and O’Malley framework for methodological review, we have developed a search strategy with content and methodological experts, and which has undergone Peer Review of Electronic Search Strategies review. A search of four scientific databases, as well as gray literature, will be performed without restriction to articles by type of COVID-19 vaccine, or country of study, and will include publications in the ten languages our team can handle. Bi-monthly search alerts based on the search strategy will be generated.

**Results:**

The first search will result in a stand alone peer reviewed scoping review. Bi-monthly updates will be posted on a pre-print server. Depending on the volume of literature, these updates will be synthesized and submitted for peer-review at 6 and/or 12 months.

**Conclusion:**

COVID-19 vaccine hesitancy amongst individuals with allergy persists, despite very low risk of serious adverse reactions. Our living scoping review, which includes multiple forms of knowledge translation, will be a rigorous way to address hesitancy.

## Background

The COVID-19 pandemic has impacted families worldwide. In the early weeks of the pandemic, we reported elevated rates of maternal anxiety and depression relative to population norms [1]. Yet, anxiety was even more pronounced amongst mothers with a child with food allergy [2] as well as those who consumed more COVID-19-related news [3]. Indeed, the sheer volume and, the misinformation about COVID-19, has contributed to the World Health Organization’s description of an “infodemic” [4]. Unfortunately, research suggests that the propagation and consumption of misinformation online is associated with greater vaccine hesitancy [5], which has been described as “the next challenge in the fight against COVID-19” [6]. Moreover, reports of allergic reactions to the COVID-19 vaccines have been documented [7], which may also contribute to hesitancy [8]. When reports of anaphylactic reactions triggered by the mRNA COVID-19 vaccine were first reported by patients, it was suspected, but not confirmed, that one of the triggers for these reactions was polyethylene glycol, or PEG [7, 9]. Polyethylene glycols consist of several polymers which are present in many medical and industrial products, including pharmaceuticals, cosmetics, and food products [10]. It should be noted, however, that PEG compounds are not uniform as they differ in molecular weight across products. PEG compounds can also differ in their structure as many drugs and medical devices contain PEG-derivatives like polysorbates, poloxamers, and PEG castor oils [10]. Although PEG is nearly ubiquitous in personal hygiene products and medications, pre-COVID reports of PEG-triggered anaphylaxis are rare, at 37 cases globally between 1977 and 2016 [10]. As more evidence is accumulated, the role of PEG is minor, if any, in the development of allergic reactions to vaccines [11, 12]. If PEG allergy remains a concern, despite the rarity of reactions, viral vector COVID-19 vaccines that do not contain PEG are available, such as the Johnson & Johnson vaccine and the AstraZeneca vaccine [13, 14].

Despite the low likelihood that the COVID-19 vaccine will trigger an allergic reaction [15–17], we [18] and others [19, 20] have reported that families with allergy remain vaccine hesitant due to concerns of COVID-19-vaccine-triggered anaphylaxis. A meta-analysis published in January 2022, of highly heterogeneous studies on the association between anaphylaxis and COVID-19 vaccines provides very modest evidence of a slight increase in odds of patient-reported anaphylactic reactions post-administration of an authorized vaccine amongst those aged 18–85 years, and particularly amongst females [21]. Notably, mRNA vaccines were associated with the lowest risk of anaphylaxis. The reported increase of anaphylaxis amongst women was attributed to hormonal regulation [21], an observation that may align with sex-specific reactions to other vaccines described elsewhere [22]. Whereas any type of vaccination carries risk, the authors of this recent meta-analysis failed to contextualize the risk of anaphylaxis associated with the COVID-19 vaccine, or the minimal increased risk of anaphylaxis in relation to the risk of COVID-19 complications. Of equal concern is the role of gender. Women, compared to men, make between 80 and 90% of healthcare decisions for their families, and are more likely to act as caregivers [23–25]. This caregiver role has been described amongst families managing a child’s food allergy, with 14% of mothers but no fathers reporting career limitations due to food allergy [26]. The increase in risk of anaphylaxis among women unequivocally affects the way mothers provide care and management to their children’s allergies. These observations, coupled with the COVID-19 infodemic, has underscored the need for a comprehensive, regularly updated review of literature surrounding COVID-19 vaccine hesitancy amongst those managing allergy.

The synthesis of individual research studies and interpreting them in a global context is an essential component of knowledge creation, and in turn, will contribute to knowledge translation [27]. To this end, the first objective of this scoping review is to investigate the peer-reviewed and grey literature on COVID-19 vaccine hesitancy and allergic disease and/or allergic reactions following a COVID-19 vaccine, that will be regularly maintained and updated as new evidence is published. The second objective is to inform knowledge translation activities resulting from this scoping review.

## Methods and analysis

For the aims of the proposed project, a scoping review is preferable to a systematic review as the former is useful when examining emerging evidence when there is a lack of clarity as to what more specific questions may be valuably addressed in a systematic review [28], and in which we have documented expertise [29–32].

## Inclusion and exclusion criteria

We will not restrict articles by type of COVID-19 vaccine or by country of study. Grey literature searches will be restricted to those published languages spoken by the research team (Table 1).

**Table 1:** Languages which the project team can currently handle

Language	
English	
French	
Spanish	
Swedish	
German	
Filipino	
Hebrew	
Bosnian	
Croatian	
Serbian	

## Concept

In keeping with the Arksey and O’Malley framework for methodological reviews [33], searches and screening will be independently but concurrently performed by trained staff (initials blinded for review). Articles will be included based on consensus. In the event of conflicts, the study lead (initials blinded for review) will guide a decision.

## Information sources

Four scientific databases (CINAHL, PsycINFO, Medline, Embase) will be searched, but without restrictions to patient age or language of publication. Due to the nature of the topic, the literature will be exclusively from 2020 onward.

Grey literature sites will include, but are not limited to websites of the Canadian Society of Allergy and Clinical Immunology, American Academy of Allergy, Asthma and Immunology, European Academy of Allergy and Clinical Immunology, and World Health Organization.

## Initial search strategy

The original search will be guided by (initials blinded for review), approved by the study lead (blinded for review), then peer-reviewed by a librarian colleague, per Peer Review of Electronic Search Strategies guidelines [34]. Sample scoping review search terms are presented in Table 2.

**Table 2:** Search strategy

Database: Ovid MEDLINE(R) and Epub Ahead of Print, In-Process, In-Data-Review & Other Non-Indexed Citations and Daily <1946 to December 14, 2021>
Search Strategy:
1 exp covid-19 vaccines/ or exp Coronavirus/ or Coronavirus Infections/ or COVID-19/ (144410)
2 (coronavir* or corona vir* or betacorona* or OC43 or NL63 or D614G or 229E or HKU1 or hcov* or ncov* or covid* or sarscov* or sars cov* or sarscoronavir* or sars coronavir* or 2019ncov* or 19ncov* or novel cov* or 2019novel cov* or longcovid* or postcovid* or postcoronavir* or postsars*).mp. (229420)
3 (COVID-19 or SARS-CoV-2).rx,px,ox,rn 4 (COVID-19 or COVID-19 serotherapy or ORF7b protein, SARS-CoV-2 or ORF6 protein, SARS-CoV-2 or ORF8 protein, SARS-CoV-2 or pediatric multisystem inflammatory disease, COVID-19 related or envelope protein, SARS-CoV-2 or ORF7a protein, SARS-CoV-2 or spike protein, SARS-CoV-2 or ORF3a protein, SARS-CoV-2 or severe acute respiratory syndrome coronavirus 2 or membrane protein, SARS-CoV-2 or ORF1ab polyprotein, SARS-CoV-2 or nucleocapsid protein, Coronavirus or COVID-19 vaccine).os,ps,rn,rs. (15173)
5 (variant* adj2 (alpha or beta or delta or gamma or kappa or lambda or mu or omicron or "b.1.1.7" or "b.1.351" or "p.1" or "b.1.617.2" or "b.1.1.529" or "c.37" or "b.1.621" or india or indian or south africa* or uk or british or english or brazil*)).mp. (5611)
6 ("mrna 1273" or elasomeran or cx024414 or "cx 024414" or "tak 919" or mrna1273 or tak919 or m1273 or "m 1273" or ad26covs1 or "ad26 cov2 s" or "jnj 78436735" or jnj78436735 or bnt162* or "bnt 162" or "bnt 162c2" or "bnt 162b2" or "pf 07302048" or pf07302048 or abdavomeran or pidacmeran or vaxzevria or spikevax or comirnaty or tozinameran or chadox1 or "chadox 1" or azd1222 or "azd 1222" or bibp or bbibpcorv or bbibp corv or sputnik or rad26-s or gamcovidvac or gam covid vac or gam kovid vak or coronavac or picovacc or covaxin or bbv152* or "bbv 152" or "bbv 152a" or "bbv 152b" or "bbv 152c" or ad5ncov or "ad5 ncov" or convidecia or pakvac or wibp or wibpcorv or abdala or cigb66 or "cigb 66" or epivaccorona or epi vac corona or auroracov or aurora cov or zifivax or zf2001 or soberana or finlayfr2 or "finlay fr 2" or pasteurcovac or qazcovid* or qazvac or nuvaxovid or covovax or nvxcov2373 or "nvx cov2373" or tak019 or "tak 019" or kconvac or covivac or coviran or covidful or mvccov1901 or mvc cov1901 or zycovd or "zycov d" or fakhravac or covax19 or "covax 19" or spikogen or ag0302 or "ag 0302" or convidicea or ad5ncov or ad5 ncov or "ad 5 ncov" or ganulameran or pittcovacc or nadorameran or zorecimeran or cvncov or reluscovtogene or ino4800 or "ino 4800").mp. (2999)
7 or/1-6 (238971)
8 exp vaccination refusal/ or antivaccination movement/ (819)
9 (exp immunization/ or exp immunization program/ or exp vaccines/) and (exp decision making/ or risk assessment/ or exp safety/ or trust/ or exp "patient acceptance of health care"/) (9680)
10 ((vaccin* or immuniz* or immunis*) adj6 (avoid* or doubt* or fear* or confiden* or concern* or hesita* or refus* or reject* or delay* or uncertain* or choice* or choos* or decid* or decis* or undecide* or barrier* or obstacle* or deter* or readiness or accept* or uptake or complian* or noncomplian* or risk*)).mp. (27628)
11 (antivac* or anti vac* or antivax* or anti vax*).mp. (1034)
12 or/8-11 (33989)
13 exp hypersensitivity/ or "allergy and immunology"/ or exp allergens/ or "insect bites and stings"/ or immunotherapy/ or exp desensitization, immunologic/ (433877)
14 (hypersensi* or hyper sensi* or hyperresponsiv* or hyper responsiv* or allergy or allergi* or allergen* or intoleran* or drug eruption* or dress syndrome* or erythema nodosum or cont?usiform* or nicolau* or serum sickness or stevens-johnson or lyell* or erythema multiforme or toxic epidermal necrolys* or photoallerg* or photo* contact dermati* or photo* dermati* or toxicodendron dermati* or rhus dermati* or vernal conjuncti* or vernal keratoconjuncti* or spring conjuncti* or summer bronchitis or anaphyla* or eczema or fancier* lung* or breeder* lung* or farmer* lung* or hoigne* or hay fever or hayfever or pollinos* or urticaria or hive or hives or immune fever or arthus or atopy or atopia or atopic or contact reaction or fpies or food protein induced enterocolitis or crossreacti* or cross reacti* or eoe or immunotherap* or scit or desensiti* or de-sensiti* or hyposensiti* therap*).mp. (553243)
15 ((chemical or drug* or medication* or food* or environmental or pollen* or contact or mold or mould or ragweed or hay* or dust or dander or pet or dog or cat or egg* or milk* or dairy or peanut* or nut* or cashew* or almond* or walnut* or hazelnut* or pecan* or pistachio* or coconut* or nutmeg* or fish or shellfish or seafood or crustacean* or wheat or barley or soy* or sesame or sunflower* or poppy* or mustard* or insect* or additive* or dye* or sulphite* or bee or bees or wasp* or arthropod* or hymenopter* or vespid* or cockroach* or pine*) adj4 sensitiv*).mp. (27226)
16. ((drug* or medica*) adj3 (dermatitis or morbiliform* or morbilliform* or exanthem*)).mp
17. (eosinophil* adj2 (esophagiti* or oesophagiti*)).mp
18 or/13-17 (763715)
19 7 and 12 and 18 (128)
20 limit 19 to yr="2019 -Current" (121)
***************************
237

## Screening

After the initial search, all citations will be uploaded into Rayyan [35]. Trained student research assistants will de-duplicate the search. Thereafter, they will independently perform title and abstract screening, noting which studies are to be excluded or included from the full text screening. This process will be blinded, such that each screener does not have access to whether the other screener has decided to include or exclude an article based on the title/abstract.

After the title and abstract screening is complete, full texts will be uploaded to Rayyan [35]. Full text screening will follow the same process as for the title and abstract screening described above. Decisions whether to exclude (and the reason for exclusion) or include the full text in the review will be based on the inclusion criteria. Once screeners have independently screened the full texts, the results will be unblinded. Screeners will meet to discuss any conflicts, and in the event they cannot agree whether an article should be included, they will defer to the study lead or her designate.

For articles that are published in languages other than English, the decision to exclude or include an article will be at the discretion of the team member(s) who comprehends that language. Screeners will direct the article(s) to team members who read the particular language.

For all included articles, data will be extracted into tables. The tables, at a minimum, will include the study’s title, authors, year of publication, country of the participants, aims, sample size, study methodology, outcome measures, and key findings. At the point of extraction, the student research assistants will be asked to extract all information that may be relevant to the review. A flow chart of the search process will also be included in the initial scoping review, per the structure provided by the Preferred Reporting Items for Systematic reviews and Meta-Analyses extension for Scoping Reviews [36].

## Bi-monthly update

A bi-monthly search alert based on the search strategy will be generated by health sciences librarian (initials blinded for review). The student research assistants assigned to this project will be tasked with screening these articles and assessing if they should be excluded or included. Conflicts between the student research assistants regarding the inclusion of particular articles will be solved through discussion. If the student research assistants cannot agree, the study lead or her designate will be asked to make the final decision.

## Patient engagement

Patient partners will be consulted at all stages of this study, from design to interpretation of the results. Our patient partners are also committed to sharing the findings of this review within their patient partner network. Additionally, the results of this review will be shared on social media platforms, including our lab website; and will be used to inform future webinars hosted by our research group.

## Ethics

As this is a scoping review of existing literature, no ethical approval is required. However, the overarching study has been approved by the University of Manitoba Health Research Ethics Board.

## Knowledge translation

Following preparation of the manuscript, we will post it on a pre-print server, and submit for publication. Per conditions of funding of COVID grants, this—and all resulting publications from this project—must be open access. These updates will follow the same process as described above. Bi-monthly updates will be posted on a pre-print server. Depending on the volume of literature, these updates will be synthesized and submitted for peer-review at 6 and/or 12 months.

Plain language summaries, in the form of infographics, will be posted on the study lead’s website (URL blinded for peer review) and shared widely on social media.

## Data Availability

Not applicable.

## Abbreviation

PEG: Polyethylene glycol
